# More cases, doctor? Yes please!

**DOI:** 10.1186/1757-1626-1-38

**Published:** 2008-07-16

**Authors:** Tom Jefferson

**Affiliations:** 1 Cochrane Vaccines Field, Anguillara Sabazia, Roma 00061, Italy

The scientific basis of modern clinical practice is founded on the randomised controlled trial [1]. Trials are experimental studies in which two or more selected groups (arms) which may (or may not be) "representative"of the whole population are assigned at random to one or more interventions and sometimes to an inert control (placebo) or compared to standard practice. The idea is that randomisation, if correctly applied to sufficient numbers would spread the effect of confounders equally across all arms of the trial. Randomisation creates the ultimate experimental conditions: whatever differences I observe between arms must be due to the only remaining modifiying factor, i.e. the intervention. This could be a drug, a diagnostic test, a vaccine or even an administrative procedure or clinical pathway. Trials are needed for the registration of new drugs, vaccines and major procedures, although curiously they are not necessary to register commonly used devices such as hip or knee prostheses. As a consequence of the importance of trials a whole new branch of epidemiology (let's call it trialology) was born to study trials, their design, conduct, reporting, synthesis and the implementation of their findings [2-4].

However, in addition to some very notorious woes (publication and reporting bias, piecemeal or redundant publication, spin or faking of results) trial results are seen by some practicing clinicians as being of limited relevance to their everyday practice as they embody a population-based approach to healthcare. This approach seems at odds with everyday experience in one's surgery, department or emergency room [5]. The typical participant of a trial is described in a synthetic fashion (because of reporting standards and space constraint in journals) and often does not seem to quite fit the patient we have in front of us or the context we work in. Statements such as "if you take wondermycin three times a day for five days with food you have a 75% chance of feeling better by the end of the course" are sometimes difficult to grasp and discuss. Over 70% of physicians' information needs are about diagnosis and/or treatment and over 60% of these are foreground (i.e. relating to the patient they have in front) [5]. Crucially, the time devoted to the search for the information cannot take more than 2 minutes, especially in those health systems such as the British NHS in which consultation lenght is around 7–10 minutes [6].

In reality the biggest problem with trials is their inductive basis. Induction was first systematized by Francis Bacon (1561 – 1626) in his "Novum Organum". Induction is the process by which after repeated observations leading to the same conclusions I make a generalised statement which forms the basis of knowledge and sometimes links cause and effect. For example if I get into my car every morning and turn the ignition key and the engine starts every time, I soon conclude (wrongly) that the motion of turning the key is inexorably linked to the engine starting up. However if my battery is flat, the starter motor is broken or the engine is blown because of my lack of care the cause and effect link is broken. Equally, in a well-conducted trial I induce that whatever I have observed in my carefully collected sample of participants will also happen in all similar people across the planet. Here I have two levels of generalisation: a first level from my sample to the whole population (this is called abstraction). The second level (analytical generalisation) is when I generalise from experimental findings to theory [7,8]. This idea is based on the concept of the uniformity of nature which the scottish philosopher David Hume (1711 – 1776) correctly pointed out as simply non-existant ("An universal statement asserting the validity of the principle of induction is here inferred from a number of singular observation statements. Thus this argument is inductive. This is "the problem of induction") [9]. In other words the general statement on the likely effects of wondermycin is justifed by past and current perfomance of wondermycin. This is induction justifying itself. The problem of induction has been only partly addressed by the use of statistical probabilism, as embodied in the hypothetical statement on wondermycin already quoted. In reality, despite our use of probability theory and all its paraphernalia of curves, tests and confidence intervals the problem of induction remains. No two patients are alike and no two sets of circumstances are absolutely similar. This is why trials and all other population-based studies, even if we had the whole picture of wondermycin perfomance (which we know is very unlikely, especially for new drugs) can only give us a very broad steer in our eveyday practice [10]. The great mathematician-philospher turned peace campaigner Bertrand Russel (1872 – 1970) mocked induction and its limits with his famous story of the inductivist turkey [11,12].

"The turkey found that, on his first morning at the turkey farm, that he was fed at 9 a.m. Being a good inductivist turkey he did not jump to conclusions. He waited until he collected a large number of observations that he was fed at 9 a.m. and made these observations under a wide range of circumstances, on Wednesdays, on Thursdays, on cold days, on warm days. Each day he added another observation statement to his list. Finally he was satisfied that he had collected a number of observation statements to inductively infer that "I am always fed at 9 a.m.". However on the morning of Christmas Eve he was not fed but instead had his throat cut.

Thus the inductivist turkey ended in the pot.

If we cannot rely (at least completely) on trials to give use guidance in everyday life can we then rely on non-randomised studies? After all, these formal studies are thought to take place in conditions which are closer to everyday life. Anyone who has critically appraised large numbers of these studies knows that reporting and design problems are even worse than in trials. Is anyone not convinced by this statement? Take a second look at the last five case-control studies you have read. How many of these specified that the choice and analysis of cases and controls was done by a researcher blind to exposure status of both? This is an absolute pre-requisite in any case control study to minimise the risk of observer bias. Some of the international efforts to minimise the risk of publication bias of trials (such as the introduction of prospective registration) have never even been mentioned for non-randomised studies. In addition the problem of induction (which is still present in these studies) is made worse by the murky play of confounders. The late Professor Geoffrey Rose used to repeat the aphorism that you can adjust for the confounders you know or guess at, not for the ones you do not know.

So how can we get some guidance for our patient action plan which is more likely to be predictive than a wondermycin registration trial of what will actually happen to our patient once I give him or her the drug [12]?

The obvious answer seems to me to be other people's practical experience embodied in case reports. Up to now case reports have been considered a backwater for old fogies and experts of the exotic. However, reports of everyday problems and solutions are more likely than anything else in biomedical science to approximate and recreate the conditions we are in. Remember: Florey first demonstrated the effects of penicillin on a case series of 8. My statement on the use of case reports has two provisos.

First, cases need to be available in huge numbers to be likely to inform our actions in the millions of different situations of everyday clinical practice. This collection should be seen as an udertaking to rival the human genome project or the setting up and development of the Cochrane Collaboration. As the latter, it should be an open ended effort.

Second, the casebook (which is more likely to end up representing a casebank) needs to be housed in an easily accessible virtual building and the search facilities need to reflect the time-constraints of everyday life. A statement such as "Excuse me madam or sir, would you mind waiting outside for 3 hours while I search the net for a case similar to yours?" would not be very popular with busy practioners or bewildered patients. A variety of search engines can be constructed some even using revolutionary methods as self-organising maps (a method taken from neural network theory).

Both these provisos entail effort, standardisation of reporting formats and commitment of ever increasing numbers of practioners and perhaps patients. However, think of the practical benefits. What if we could access the case book of William Osler or even of my old family doctor who died 20 years ago? How many lessons would we learn or re-learn? Let's transform this vision into a reality. What better challenge can there be for a new journal?
